# Conférence COP27 sur le changement climatique: des mesures d’urgence sont nécessaires pour l’Afrique et pour le monde entier^*^

**DOI:** 10.26633/RPSP.2022.211

**Published:** 2022-12-15

**Authors:** Lukoye Atwoli, Gregory E Erhabor, Aiah A Gbakima, Abraham Haileamlak, Jean-Marie Kayembe Ntumba, James Kigera, Laurie Laybourn-Langton, Bob Mash, Joy Muhia, Fhumulani Mavis Mulaudzi, David Ofori-Adjei, Friday Okonofua, Arash Rashidian, Maha El-Adawy, Siaka Sidibé, Abdelmadjid Snouber, James Tumwine, Mohammad Sahar Yassien, Paul Yonga, Lilia Zakhama, Chris Zielinski

**Affiliations:** East African Medical Journal East African Medical Journal; West African Journal of Medicine West African Journal of Medicine; Sierra Leone Journal of Biomedical Research Sierra Leone Journal of Biomedical Research; Ethiopian Journal of Health Sciences Ethiopian Journal of Health Sciences; Annales Africaines de Médecine Annales Africaines de Médecine; Annals of African Surgery Annals of African Surgery; University of Exeter Exeter UK University of Exeter, Exeter, UK; African Journal of Primary Health Care and Family Medicine African Journal of Primary Health Care and Family Medicine; London School of Medicine and Tropical Hygiene London UK London School of Medicine and Tropical Hygiene, London, UK; Curationis Curationis; Ghana Medical Journal Ghana Medical Journal; African Journal of Reproductive Health African Journal of Reproductive Health; Eastern Mediterranean Health Journal Eastern Mediterranean Health Journal; Mali Médical Mali Médical; Journal de la Faculté de Médecine d’Oran Journal de la Faculté de Médecine d’Oran; African Health Sciences African Health Sciences; Evidence-Based Nursing Research Evidence-Based Nursing Research; East African Medical Journal East African Medical Journal; La Tunisie Médicale La Tunisie Médicale; University of Winchester Winchester UK University of Winchester, Winchester, UK.

## Les nations riches doivent renforcer le soutien pour l’Afrique et les pays vulnérables en faisant face aux effets passés, présents et futurs du changement climatique

Le rapport en 2022 du Groupe intergouvernemental d’experts sur l’évolution climatique (GIEC) dresse un sombre tableau pour l’avenir de la vie sur terre, caractérisé par l’effondrement des écosystèmes, l’extinction d’espèces et les dangers climatiques tels que les canicules et les inondations.^1^ Tout ceci est lié aux problèmes de santé physique et mentale, avec des conséquences directes et indirectes sur l’augmentation de la morbidité et de la mortalité. Pour éviter ces effets catastrophiques sur la santé à travers toutes les régions du globe, il existe un large consensus, comme l’ont indiqué 231 journaux de santé en 2021, précisant que l’augmentation de la température mondiale doit être limitée à moins de 1,5 °C par rapport aux niveaux préindustriels.

Tandis que l’accord de Paris de 2015 souligne un cadre de mesures mondiales incorporant un soutien financier climatique pour les pays en voie de développement, ce soutien ne s’est pas encore matérialisé.^2^ COP27 est la cinquième conférence des parties à être organisée en Afrique depuis sa création en 1995. Dans la perspective de cette réunion, nous, rédacteurs de journaux de santé à travers le continent, appelons à des mesures d’urgence pour assurer que cette COP apporte enfin une justice climatique pour l’Afrique et pour les pays vulnérables. Ceci non seulement est essentiel pour la santé de ces pays mais également pour celle du monde entier.

## L’Afrique a souffert de façon disproportionnée, même si elle n’est pas à l’origine de la crise

La crise climatique a eu un impact sur les déterminants environnementaux et sociaux de la santé à travers l’Afrique, entraînant des effets dévastateurs sur la santé.^3^ Les effets sur la santé peuvent être le résultat direct de chocs environnementaux et résulter indirectement d’effets liés aux circonstances sociales.^4^ Les risques liés au changement climatique en Afrique incluent les inondations, la sècheresse, les canicules, une baisse de la production alimentaire et une diminution de la productivité de la main-d’œuvre.^5^

Les sècheresses en Afrique subsaharienne ont triplé entre 1970-1979 et 2010-2019.^6^ En 2018, des cyclones dévastateurs ont affecté 2,2 millions de personnes au Malawi, au Mozambique et au Zimbabwe.^6^ En Afrique occidentale et centrale, de graves inondations ont entraîné des fatalités et une migration forcée à cause de la perte de logement, de terrains cultivés et du bétail.^7^ Des changements dans l’écologie des vecteurs, provoqués par les inondations et les dégâts sur l’hygiène environnementale, ont conduit à une augmentation des maladies à travers l’Afrique subsaharienne, avec des incidences accrues du paludisme, de la dengue, de la fièvre de Lassa, de la fièvre de la vallée du Rift, de la maladie de Lyme, du virus Ebola, du virus du Nil occidental et d’autres infections.^8, 9^ La montée du niveau des mers réduit la qualité de l’eau, entraînant des maladies véhiculées par l’eau, y compris des maladies diarrhéiques, une cause majeure de mortalité en Afrique.^8^ Un climat extrême cause des dégâts sur l’approvisionnement en eau et en nourriture, augmentant l’insécurité alimentaire et la malnutrition, ce qui entraîne 1,7 million de morts par année en Afrique.^10^ Selon l’organisation des Nations Unies pour l’alimentation et l’agriculture, la malnutrition a augmenté d’environ 50% depuis 2012, à cause du rôle primordial que l’agriculture joue dans les économies africaines.^11^ Les chocs environnementaux et leurs répercussions nuisent aussi gravement à la santé mentale.^12^ Une estimation suggère que la crise climatique a, dans l’ensemble, détruit un cinquième du produit intérieur brut (PIB) des pays les plus vulnérables aux chocs climatiques.^13^

Les dommages causés à l’Afrique doivent être d’une préoccupation suprême pour toutes les nations. Et ce en partie pour des raisons d’ordre moral. Il est extrêmement injuste que les nations les plus touchées soient celles ayant contribué le moins aux émissions mondiales cumulées, responsables de la crise climatique et de ses effets de plus en plus graves. L’Amérique du Nord et l’Europe sont responsables de 62% de la production de dioxyde de carbone depuis la révolution industrielle, tandis que l’Afrique ne compte que pour 3%.^14^

## La lutte contre la crise climatique exige que tout le monde soit sur le pont

Ce n’est cependant pas uniquement pour des raisons d’ordre moral que toutes les nations doivent se préoccuper de l’Afrique. Les effets aigus et chroniques de la crise climatique créent des problèmes tels que la pauvreté, les maladies infectieuses, la migration forcée et les conflits qui s’étendent à travers les systèmes mondialisés.^6, 15^ Ces répercussions concernent toutes les nations. Covid-19 a permis une prise de conscience de ces dynamiques mondiales et ce n’est pas une coïncidence si les professionnels de santé ont activement participé à l’identification et à la réponse aux conséquences de risques sanitaires systémiques croissants. Mais les leçons tirées de l’épidémie de Covid-19 ne doivent pas se limiter au risque pandémique.^16, 17^ Au contraire, il est impératif que la souffrance des nations en première ligne, y compris celles d’Afrique, soit la préoccupation principale de COP27: dans un monde interconnecté, laisser des pays à la merci des chocs environnementaux crée une instabilité qui entraîne des conséquences graves pour tout le monde.

L’objectif principal des sommets climatiques reste de réduire rapidement les émissions, de sorte que les augmentations de la température mondiale soient maintenues en dessous de 1,5 °C. Ceci limitera les dégâts. Mais pour l’Afrique et d’autres régions vulnérables, ces dégâts sont déjà graves. Atteindre la cible promise d’une finance climatique de 100 milliards de dollars par année est désormais essentiel à l’échelle mondiale, si nous voulons éviter les risques systémiques de laisser des sociétés en crise. Ceci peut s’accomplir en assurant que ces ressources se concentrent sur l’accroissement de la résilience face aux effets inévitables actuels et futurs de la crise climatique, tout en soutenant les nations vulnérables dans la réduction de leurs émissions de gaz à effet de serre : une parité d’estime entre adaptation et limitation. Ces ressources doivent être accordées par l’intermédiaire de subventions et non pas d’emprunts, et être augmentées de manière urgente avant la période considérée de 2025. Elles doivent placer la résilience du système de santé en première ligne, puisque les crises combinées causées par la crise climatique se manifestent souvent par de graves problèmes de santé. Le financement de l’adaptation sera plus rentable que de se reposer sur les secours en cas de catastrophe.

Des progrès ont été faits pour l’adaptation en Afrique et dans le monde entier, y compris les systèmes et les infrastructures d’alerte précoce pour se défendre contre des circonstances extrêmes. Mais les nations en première ligne ne reçoivent pas de compensations pour les effets d’une crise dont elles ne sont pas la cause. Ceci est non seulement injuste, mais entraîne également une spirale de déstabilisation mondiale, puisque les pays donnent de l’argent en réponse aux catastrophes mais n’ont plus les moyens de soutenir une résilience plus importante ou de régler le problème à sa base, à savoir les réductions d’émissions. Une facilité de financement pour pertes et dommages doit maintenant être introduite, apportant des ressources supplémentaires allant au-delà de celles accordées pour la limitation et l’adaptation. Ceci doit aller au-delà des échecs de COP26, lorsque la suggestion d’une telle facilité fut réduite à « un dialogue. »^18^

La crise climatique est un produit de l’inaction à l’échelle mondiale et se traduit par un coût élevé non seulement pour les pays africains affectés de manière disproportionnée mais pour le monde entier. L’Afrique s’unit à d’autres régions en première ligne pour demander aux nations riches d’intervenir enfin, si ce n’est que parce que les crises en Afrique vont, tôt ou tard, s’étendre et engloutir tous les coins du monde, et il sera alors trop tard pour répondre avec efficacité. Si elles n’ont pas encore été persuadées par des raisons d’ordre moral, espérons que leur intérêt personnel prendra maintenant le dessus.
